# A chiral-based magnetic memory device without a permanent magnet

**DOI:** 10.1038/ncomms3256

**Published:** 2013-08-06

**Authors:** Oren Ben Dor, Shira Yochelis, Shinto P. Mathew, Ron Naaman, Yossi Paltiel

**Affiliations:** 1 Department of Applied Physics, Center for Nanoscience and Nanotechnology, Hebrew University of Jerusalem, Jerusalem 91904, Israel; 2 Department of Chemical Physics, Weizmann Institute of Science, Rehovot 76100, Israel

## Abstract

Several technologies are currently in use for computer memory devices. However, there is a need for a universal memory device that has high density, high speed and low power requirements. To this end, various types of magnetic-based technologies with a permanent magnet have been proposed. Recent charge-transfer studies indicate that chiral molecules act as an efficient spin filter. Here we utilize this effect to achieve a proof of concept for a new type of chiral-based magnetic-based Si-compatible universal memory device without a permanent magnet. More specifically, we use spin-selective charge transfer through a self-assembled monolayer of polyalanine to magnetize a Ni layer. This magnitude of magnetization corresponds to applying an external magnetic field of 0.4 T to the Ni layer. The readout is achieved using low currents. The presented technology has the potential to overcome the limitations of other magnetic-based memory technologies to allow fabricating inexpensive, high-density universal memory-on-chip devices.

## Abstract (web summary)

Most new device concepts for random-access memory are based on inorganic spin filters, which need a permanent magnet to operate. Here, the authors exploit the chiral-induced spin selectivity effect in an organic spin filter to construct a novel type of memory device, which works without a permanent magnet.

## Graphical abstract

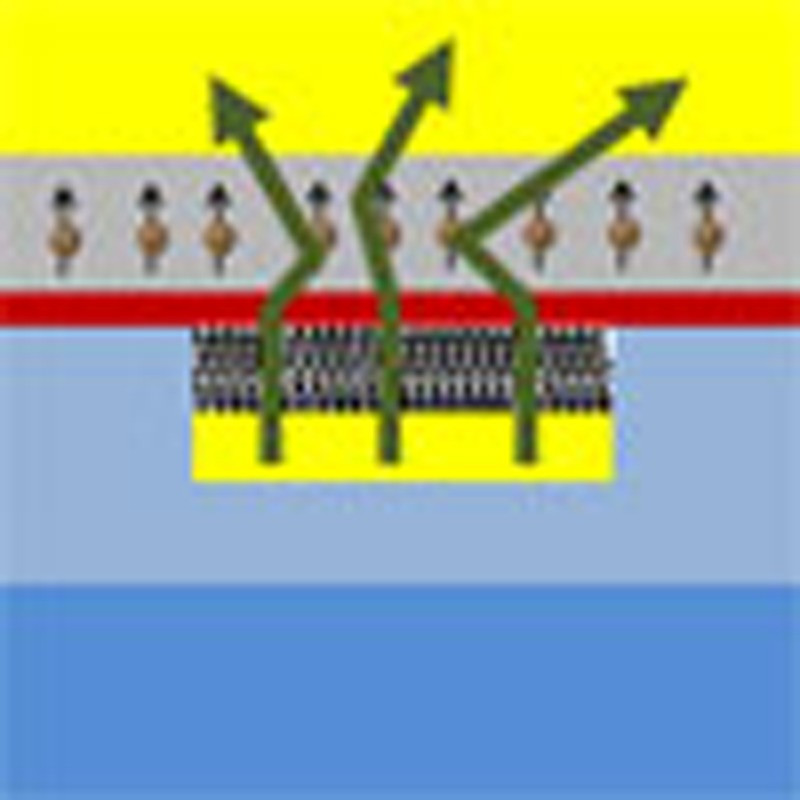

Substantial improvement in computation speed requires smaller and denser universal memory with high speed and low power consumption. Dynamic random-access memory^1^ is a type of memory that stores each bit of data in a separate capacitor within an integrated circuit. However, it has to be refreshed periodically. In static random-access memory (SRAM)^2^ the word *static* indicates that, unlike dynamic random-access memory, it does not need to be periodically refreshed, because SRAM uses a bistable circuit to store each bit. SRAM stores the data as long as the power is supplied to the system, but the data are eventually lost when the memory is not powered. This type of memory is commonly used on-chip. Flash memory is a non-volatile memory based on silicon oxide. It is inexpensive and can store data without power for 10 years, but it lacks speed.

Two possible technologies are considered most relevant for serving as universal memory: magnetic memory^3^ and spin-transfer torque memory^4^,^5^. Both are based on inorganic spin filters. In magnetic memory, a permanent magnetic layer is separated by a thin isolating layer from another ferromagnet (a free layer) that can be magnetized either parallel or antiparallel to the permanent magnet orientation. The magnetization occurs by a current loop. In spin-transfer torque memory, the device looks very similar, but here the magnetization of the ferromagnetic free layer is determined by transferring spin alignment to it, depending on the direction of the charge flow^6^,^7^.

In both cases, the devices are based on the spin-filter concept with a permanent magnet. Namely, if the free layer and the permanent magnet are aligned in parallel, the resistivity of the device is lower than when they are aligned antiparallel to each other. Some devices are based on the giant magnetoresistance effect and some on tunnelling magnetoresistance^8^,^9^. Despite their advantages, these technologies are not widely used because of technical problems involved in producing a permanent magnet on a small scale at room temperature.

In recent years, studies of electron transfer through organic molecules have shown that such molecules can introduce quantum mechanical properties, such as discreet energy levels, to the standard electronic components at ambient temperatures^10^. One such effect is electron transfer through chiral molecules which is spin selective^11^. In several studies, the chiral-induced spin selectivity (CISS) effect was established, in which chiral molecules, especially helical ones, can serve as very efficient spin filters^12^,^13^,^14^. This effect was explained theoretically in several papers that included quantitative calculations^15^,^16^,^17^,^18^.

In this study, we present a magnetic-based Si-compatible universal memory device without a permanent magnet. The magnetless spin memory technology uses the above concept for spin selectivity instead of the common ferromagnetic-based spin filters. Here, spin-selective charge transfer through a self-assembled monolayer (SAM) of polyalanine magnetizes a thin Ni layer. This change allows, in principle, the memory bit to be miniaturized, down to a single magnetic nanoparticle size.

## Results

### Device concept

**Figure 1:**
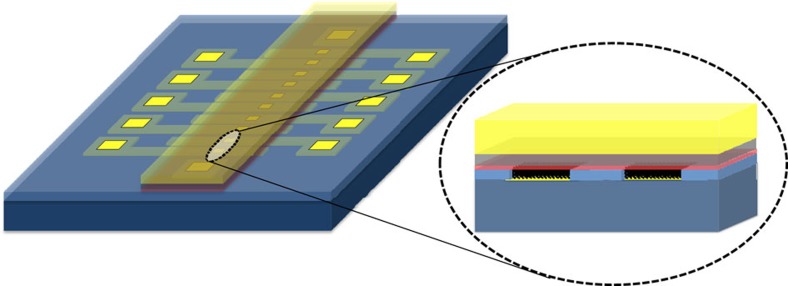
Device scheme. Schematic drawing of the device, with a top view on the left and a blowout side view on the right. The gold contacts are represented in yellow, and the thin ferromagnetic Ni is in transparent purple. The Al_2_O_3_ layer above the chiral molecules is in red. The Si is displayed in dark blue, and the SiO_2_ is displayed in light blue. The reading or writing is performed by driving the current between the two gold contacts. When current flows through the device, the electrons are polarized by the chiral molecules, and, consequently, they transfer their polarization to the Ni and magnetize it. The reading is achieved by driving a small current and measuring the change in the resistance. The resistance correlates with the magnetization of the Ni. Positive voltage in our electrode configuration is when the bottom contact receives positive voltage and the top contact is grounded.

In all our studies, we used SAMs of α-helix L-polyalanine (AHPA-L) adsorbed on gold. Figure 1 presents a top view and blowout side view schemes of the micron-sized device.

**Figure 2:**
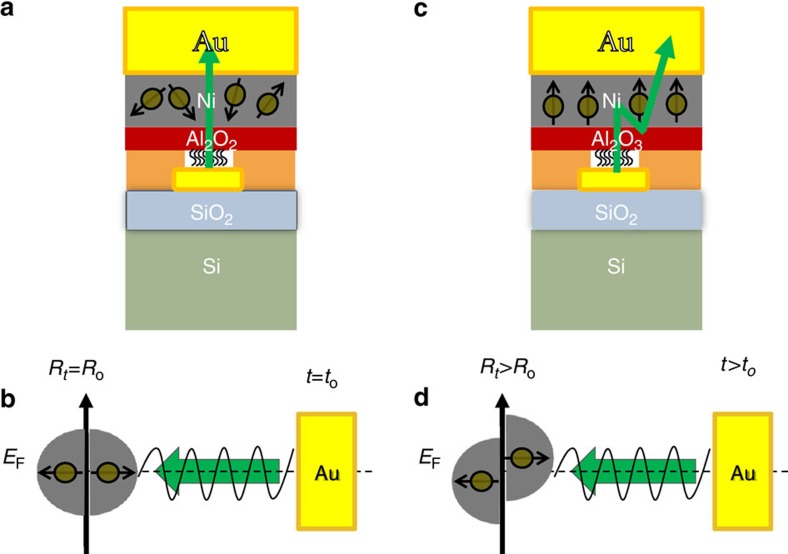
Schematic drawing of the experimental concept. (**a**) Initially, low resistance is measured as spins parallel to the electron's velocity (green arrow) are transferred through the chiral layer into the non-magnetized Ni. (**b**) The corresponding Fermi energy in Ni. The directional spins are injected into Ni near *E*_F_. This is a state of low resistance. (**c**) Further injection of spin-polarized electrons increases the Ni magnetization, after which the Ni magnetization is aligned parallel to the injected spins. (**d**) After having been magnetized, the majority and minority spin density of states are split near *E*_F_, and the majority spin states inside the Ni are almost completely filled; therefore, the resistivity increases as the injection of majority spins continues.

**Figure 3:**
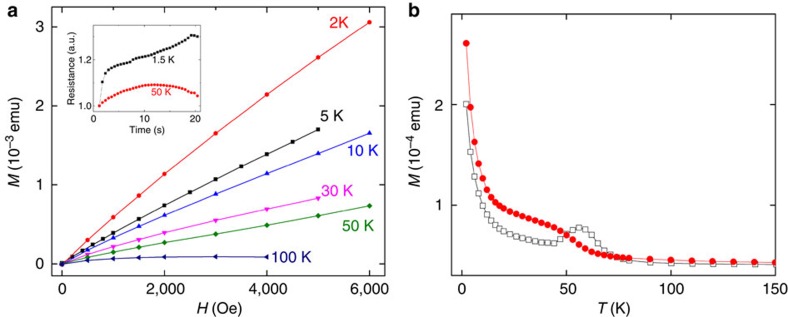
Magnetization dependence on temperature. (**a**) Perpendicular magnetization of the device with a thin Ni layer, as a function of an external magnetic field up to 0.5 T, at different temperatures. The magnetization is stronger at 2 K as compared with 50 K. The inset shows the resistance change in the sample under a constant voltage of −2.5 V and 0 T external magnetic field at 1.5–50 K. (**b**) ZFC (empty squares) and FC (red circles) perpendicular magnetization (out-of-plane) of the device measured at 500 Oe.

The concept of the experiment is explained in Fig. 2. Electrons that are ejected from the gold substrate are transferred through the right-handed chiral AHPA-L into the Ni layer, and finally to the collector gold electrode. As the transmission through AHPA-L is spin selective, as predicted by the CISS effect, the electrons entering the nickel have mainly one spin orientation, depending on the chirality and the direction of the velocity of the electrons. As our results indicate (see below), in this specific case, the spin is oriented mainly parallel to the electrons' velocity. The electrons passing through the nickel layer transfer spin torque to it, inducing magnetization. When magnetized, the nickel's resistivity increases for electrons having spins aligned parallel to the magnetic dipole of the nickel^19^. It is well known that the easy axis of a thin Ni layer lies in a plane^20^. Therefore, when Ni is magnetized perpendicular to the surface, the magnetic moment flips to the in-plane direction on a temperature-dependent timescale (see Fig. 3). Because in the present configuration the spin of the transferred electrons through the layer is polarized parallel to their velocity, magnetization is induced in the layer perpendicular to its surface. However, this magnetization is not stable, and the results should indicate competition between the magnetization and demagnetization processes. The long-lived magnetization in our configuration is small and is related to the large roughness of the thin ferromagnetic layer. In the present device, the roughness of the Ni is of the order of 10 nm. Thus, domains with longer and stronger perpendicular magnetization can be created^21^. The characteristics of the domains are strongly dependent on the fabrication process and vary from sample to sample.

### Magnetic measurements

Figure 3a presents the perpendicular magnetization as a function of the applied field at various fixed temperatures, as measured by the superconducting quantum interference device (SQUID) magnetometer, on the studied device. The inset shows the temperature dependence of the resistance change in the device under a constant voltage of −2.5 V (Fig. 3a inset). The resistance change, under a constant voltage, is strong at 1.5 K and weak at 50 K. For the magnetization measurements, the magnetic field is ramped to the set value and is stabilized in ‘no overshoot' mode. The magnetization takes about a minute to be measured, and three measurements were carried out to collect one data point, indicating stable magnetization for this time period. The *M–H* isotherms were measured up to 0.5 T at various temperatures, indicating that the saturation point is considerably delayed. This is mainly due to the in-plane anisotropy, which makes it difficult for the field to lift the domain magnetization out of the sample plane as well as in the presence of paramagnetic spins. Considering that the thickness of the Nickel film is 30 nm, the perpendicular magnetization measured at *H*=0.5 T and *T*=2 K is significantly larger than expected, taking into account that the out-of-plane is usually the hard direction of magnetization for the Ni films.

Above 50 K, in the absence of an external magnetic field, the domain's magnetization lies within the sample plane owing to the strong in-plane anisotropy. Hence, it is expected that in the present device, the effect of magnetization due to spin-torque transfer will be observed only at low temperatures. In addition, when the Ni becomes magnetized, the current decreases, and demagnetization competes with the magnetization process and consequently reduces the resistivity, as indicated in the insert of Fig. 3a. for 50 K. For longer periods, oscillatory behaviour is measured under constant voltage. When the resistance decreases and the current increases, the magnetization is enhanced. For a magnetized sample, the resistance increases again and the current decreases.

Figure 3b compares the magnetization measured in zero-field-cooled (*M*_ZFC_(*T*)) and field-cooled (*M*_FC_(*T*)) modes at a field of 500 Oe, applied perpendicular to the nickel film. *M*_ZFC_(*T*) and *M*_FC_(*T*) indicate that the ferromagnetic order is set around 60 K, though a fraction remains paramagnetic throughout the temperature range. Below 60 K, bifurcation is observed between the ZFC and FC magnetic curves, and the irreversibility in magnetization, *M*_FC_(*T*)−*M*_ZFC_(*T*), is temperature and field dependent. This suggests that the system has bistable magnetic states, separated by an energy barrier due to the in-plane anisotropy^22^,^23^. When the sample is cooled, in the presence of a perpendicular magnetic field, most of the domain magnetization points along the field direction and results in a high-magnetization stable state (FC). However, when the magnetic field is applied at the lowest temperature, after cooling the sample in zero field, a sizable fraction of the domain magnetization gets trapped in the sample plane, owing to the in-plane magnetic anisotropy, which results in a low-magnetization metastable state (ZFC). This trapped domain magnetization can overcome the in-plane anisotropy energy barrier either by thermal activation or by spin-torque transfer. These results are consistent with the observation that the memory effect, which we attribute to spin-torque transfer, can drive the system between the two states only below 60 K.

### Magneto-transport of the device

**Figure 4:**
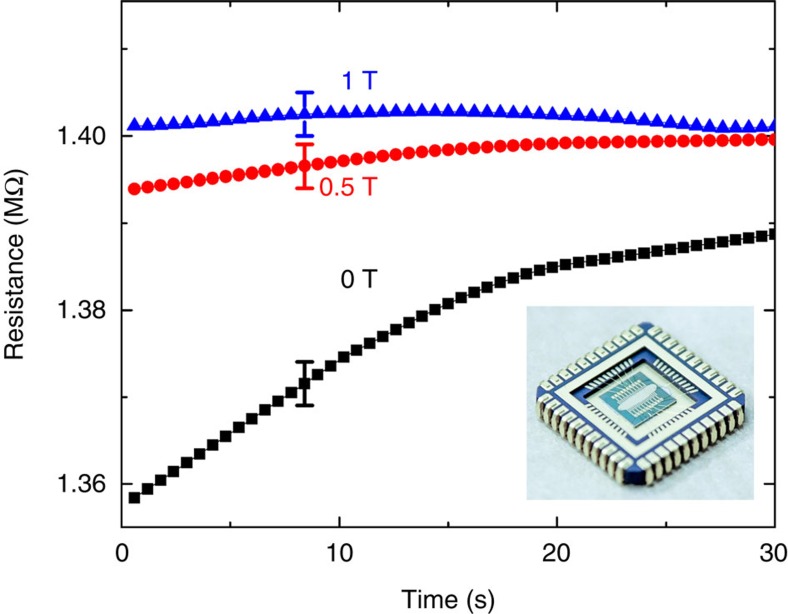
The time dependence of the device resistance at 2 K. The black square line represents the resistance increase when the current is driven through the chiral layer at a constant voltage of −2.5 V. At 0.4 T (0 A), we achieve similar resistance as the maximum resistance measured by the spin-filter effect using −2.5 V. The red circle (blue triangle) line is the same measurement under a 0.5 T (1 T) magnetic field parallel to the current direction. The inset shows an optical microscopy picture of the measured device. Error bars indicate the maximum fluctuation of the measuring device.

The black curve in Fig. 4 represents the resistance change in the nickel layer of a device without an external field at 2 K. At a constant voltage of −2.5 V, the resistance increases until a maximum value is reached. We attribute the resistance change to the magnetization of the Ni. With 30 nm thickness, the Ni demagnetization is fast and strongly depends on temperature (see Fig. 3). Therefore, two competing processes take place. One is the magnetization of the Ni domains out of plane, owing to the current transfer through the organic spin filter, and the other one is the demagnetization process due to the in-plane anisotropy and other effects such as direct tunnelling or coherent loss in the barrier. With time, the magnetization increases, and the resistance for the majority of spins increases. Above 100 K, no change in the resistivity is observed, upon applying the current, and indeed no irreversibility in magnetization was measured with the SQUID magnetometer. At 2 K, the magnetization increases with time, and consequently the resistance increases.

Applying a magnetic field (0.5 T red curve in Fig. 4) parallel to the current direction magnetizes the Ni, and therefore the resistance starts at a higher value. For 1 T, the Ni is fully magnetized, even when no current is applied, the resistance is the highest and no change in resistance is measured with time.

As the 0.5 T field is just below the saturation field required for magnetizing the Ni layer, we compared the voltage with current measurements when the sample is rotated with respect to the 0.5 T applied field. When the long axis of the chiral molecules is aligned parallel to the field and the electrons flow from the gold to the nickel, the resistance is expected to increase, as seen in Fig. 4. With our notation, 0° angle is the direction of the spins, as shown in Fig. 2b. On the other hand, if the electrons flow in the same direction and the field is rotated (180° with our notation), the magnetization of the nickel will be opposite to that of the spin transferred through the molecules. Hence, the spin flowing to the nickel represents the minority of carriers, and the resistance therefore should be smaller. When the axis of the major molecules is perpendicular to the magnetic field (90° with our notation), the spins injected are aligned perpendicular to the magnetic field, and therefore they can fill both the majority or minority spin states, and hence the current is not expected to affect the resistance, and indeed this was observed.

**Figure 5:**
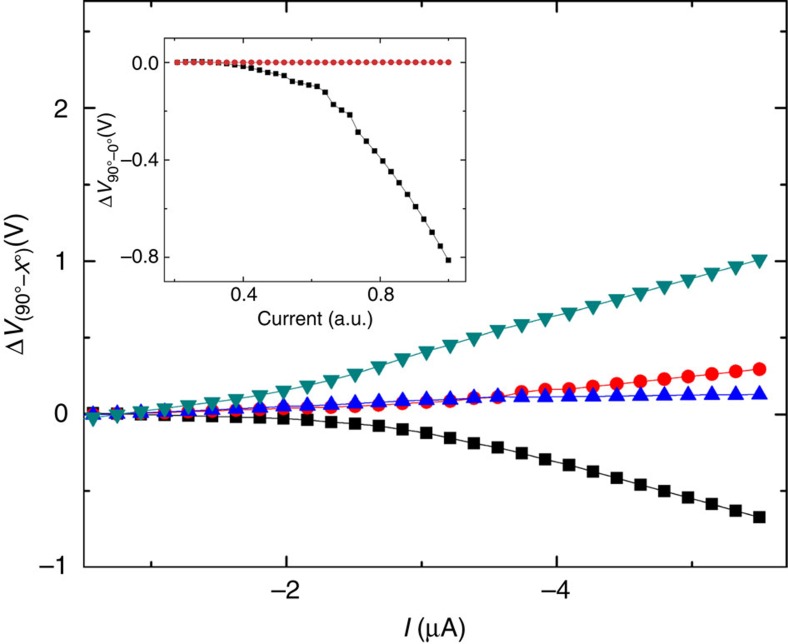
Magnetic field orientation dependence. The voltage difference, Δ*V*, between the voltages measured with the magnetic field aligned perpendicular to the surface normal (90°) and the voltage measured at different angles. All measurements were done at 35 K under a magnetic field of 0.5 T. The black squares represent the difference in voltage measured between 90° and 0°. The light-green triangles pointing down represent the difference in voltage measured between 90° and 180°. The red circles and the blue triangles pointing up show intermediate cases of 45° and 135° accordingly. The inset compares the voltage change in a reference device, without the organic molecules (red circles) and the real device with the AHPA-L molecules (black squares), measured between the 90° and 0° angles at 0.5 T magnetic field.

Figure 5 shows the voltage difference, Δ*V*, as a function of the current. Δ*V* represents the difference in the voltage measured at the 90° configuration and that measured at other angles between the magnetic field and the molecules' major axis. All measurements were performed at 35 K at a field of 0.5 T. The black squares represent the difference in voltage measured between the 90° and the 0° angle. As explained above, subtracting the voltage measured at 0° from that measured at 90° should result in a negative slope when the current increases. This is because the resistance is larger when the magnetic field is parallel to the current flowing through the molecules. On the other hand, a positive slope for an absolute current increase is expected when the voltage measured at 180° is subtracted from that measured at 90°. For the 45° and 135° cases only, a small change in resistance is measured as compared with the 90° case; nevertheless, the slope is positive for 45° and negative for 135°, as expected. We attribute the small change, for the 45° and 135° cases, to pinning of the Ni magnetization to the sample plane. The inset of Fig. 5 compares the voltage change under 0.5 T and a 0° angle between a reference device without the organic molecules (red circles) and the real device (black squares). In the reference, without the chiral molecules, no change in voltage is measured when the angle of the sample is changed with respect to the field direction.

Interestingly, using the present experimental configuration, the magnetization of the thin nickel film is in the same direction independent of whether the current flows from the gold substrate to the nickel or *vice versa*. With the CISS effect, the spin polarization is related to the electrons' velocity; hence, when the electrons' velocity reverses direction, so does the spin of the transferred electrons. Therefore, while in one direction of current (electrons transported through the chiral molecules to the Ni) the majority of spins are injected, reversing the current (electrons transported from the Ni through the chiral molecules' filter) removes the minority of spins from the Ni layer, leaving the Ni magnetized by the majority. Consequently, in our configuration, the writing can be done using both voltage directions. The reading is performed by driving a small current and monitoring the resistivity. The memory can be erased at high voltages. When the applied voltage is higher than the barriers for the transport of both spins through the chiral layer, the selectivity for spin is reduced^13^. When the voltage applied is increased by another order of magnitude, both types of spins are injected in similar rates to the Ni layer, and therefore demagnetize the Ni.

**Figure 6:**
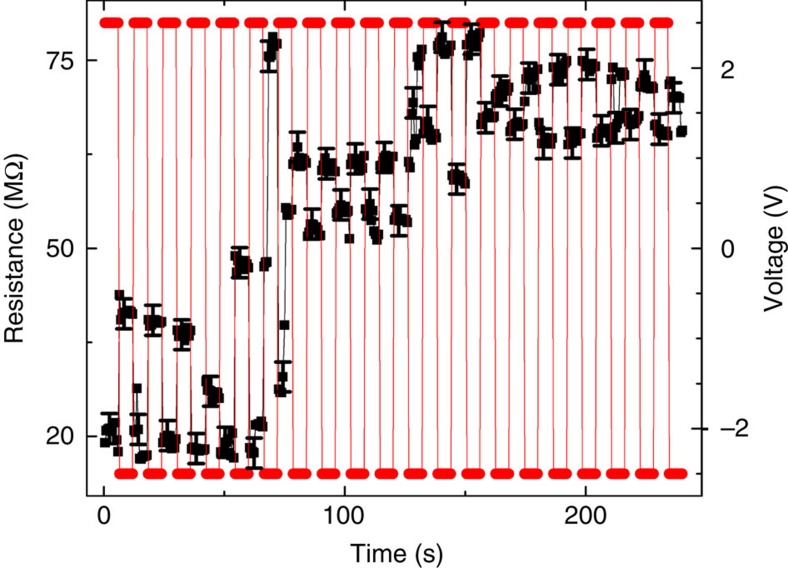
Magnetization for different current directions. Slow Ni magnetization effect at 47 K under a 0 T external magnetic field, using a sequence of +2.5 and −2.5 V voltages to drive the current in both directions. The voltage sequence is represented by red circles; the resistance of the device is represented by black squares. The magnetization of the sample increases with time. Error bars indicate the maximum fluctuation of the measuring device.

**Figure 7:**
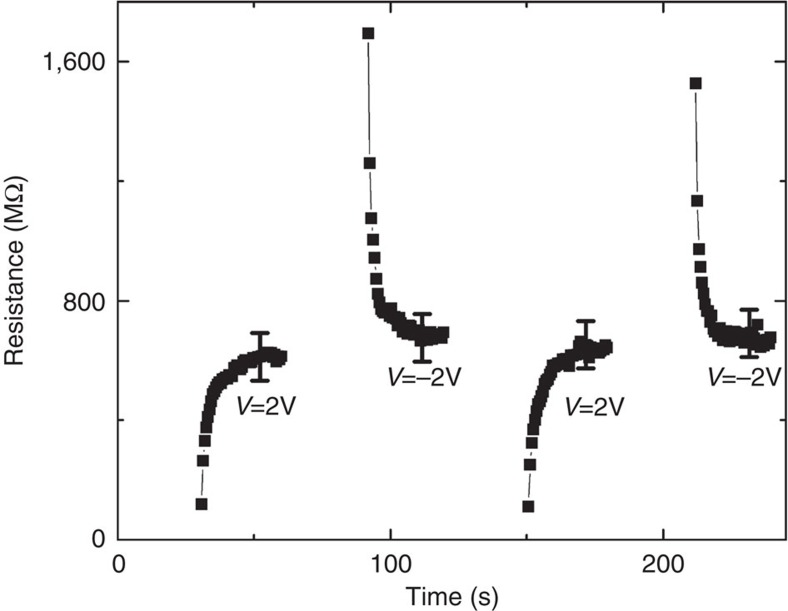
Memory effect. Writing the memory at −15 V for 30 s (empty spaces between +2 and −2 V measurements) and reading at lower voltages of +2 and −2 V. The initial resistance is high for one direction of current and low for the opposite direction of current. Measurements were performed at 1.5 K and under a 0 T external magnetic field. Error bars indicate the maximum fluctuation of the measuring device.

Figure 6 shows the Ni magnetization effect using a sequence of +2.5 and −2.5 V voltage pulses to drive the current in both directions at 47 K. The voltage sequence is represented by red circles, and the resistance of the device is represented by black squares. Clearly, the resistance increases with time. At 47 K, the thermal energy is comparable to the effective anisotropy energy barrier (see Fig. 3b), and hence the thermal activation can switch the domain magnetization between the bistable states. This explains the large noise of the measurements and the changing difference between the positive and negative voltages. The difference between the resistance of −2.5 and +2.5 V at subsequent time intervals is related to the asymmetry of magnetization when the current is driven in opposite directions. This effect will be presented more clearly in Fig. 7.

### Memory effect

The memory effect is demonstrated in Fig. 7. We write and magnetize the Ni at −15 V and read it using lower voltages of −2 or 2 V. For 2 V, we first measure the transfer of the minority spin carriers; therefore, low resistance is observed that increases with time; for −2 V, we start at high resistance by injecting the majority of spins. Owing to strong demagnetization of the Ni, the memory survives for only a few seconds. This writing and reading is repeatable, and there is more than an order of magnitude difference between the resistivity measured for −2 and +2 V during the first few seconds.

## Discussion

The technology presented here is Si compatible; therefore, a device could be miniaturized to the limit achieved by Si technology (22 nm today). However, the permanent magnetic layers in the memory device can be miniaturized only to a size of about 100 nm because of material limitations^24^. Recently, we have developed selective adsorption techniques to address this problem^25^. By combining the two processes, it is possible to fabricate the described memory device with many ports of chiral molecules that inject spins into the paramagnetic layer. By choosing several of the ports to inject the spin up and several ports to inject the spin down, a memory operating at a high base can be realized. Also, with two opposite ports simple XOR logic is achievable. Driving the current through both ports results in a non magnetize sample, the same as the zero current case. Using one port only will magnetize the sample. Lastly, by changing the Ni layer thickness to other dimensions or by replacing the ferromagnetic material, longer memory times could probably be achieved at higher temperatures. For example, using 10 nm ferromagnetic nano particles with easy axis in the direction of magnetization can help enhance the demagnetization time by several orders of magnitude.

Using the CISS effect with a SAM of AHPA-L and standard Si technology, we were able to magnetize a Ni layer. Similar magnetization can be achieved using an external magnetic field of 0.5 T. Furthermore, we demonstrated a memory effect at low temperatures. The device presented here is obviously only a preliminary indication of the ability to produce spin-based devices with no permanent magnet. Using the multi-port chiral configuration, the technology presented here has the potential to overcome the limitations of other magnetic-based memory technologies and could render possible the fabrication of inexpensive, high-density universal memory-on-chip devices.

## Methods

### Sample preparation

The device was realized using seven major steps (see Fig. 1): (a) evaporating the gold bottom contact, (b) growing a 500-nm plasma enhanced chemical vapor deposition SiO_2_ layer, (c) opening 40 × 50 μm^2^ windows in the SiO_2_ layer, (d) selective adsorption of the chiral molecules on top of the gold bottom contact, (e) evaporation of a 4–7-nm thick Al_2_O_3_ tunnel barrier, (f) evaporation of a 30-nm thick Ni ferromagnetic layer and (g) evaporation of the top gold contact.

### Self-assembly of the chiral layer

The organic AHPA-L chiral layer was prepared in three steps. First, the devices were left in absolute ethanol for 20 min before they were immersed into a 1-mM ethanol solution of the organic molecule overnight. This procedure allows the self-assembled molecules to form a homogeneous, closely packed single layer of molecules. In the second step, an excess of the organic molecules is removed from the surface by washing the sample with ethanol several times. In the last step, the samples are dried under nitrogen.

### Measurement systems

The measurements were conducted using a closed loop Oxford spectromagPT system at temperatures ranging from 1.5 to 300 K and magnetic fields ranging from 0 to 5 T. The current is driven through the molecules in both directions, between top and bottom contacts, using positive or negative voltage. Positive voltage in our electrode configuration is when the bottom contact receives positive voltage and the top contact is grounded. For the negative voltage, the majority of electron spins are injected into the Nickel layer, and for the positive voltage, the minority of electron spins are removed from the Nickel layer. To achieve higher statistics, many windows were opened in each device, and the transport behaviour was compared in all the windows. The measurements were repeated many times, and the results were averaged and smoothed over all the measurements. The Al_2_O_3_ tunnel barrier ensures that the evaporated Ni does not diffuse and contact the gold substrate. Hence, it solves one of the major problems in producing vertical organic–inorganic electronic devices that are based on a self-assembled process. The magnetic measurements were carried out on a Quantum Design SQUID magnetometer.

## Author contributions

O.B.D. performed the experiments and analysis. S.P.M. preformed the magnetization measurement. S.Y. helped in all aspects related to the chemical process needed to realize the devices. O.B.D., Y.P. and R.N. analysed the data and developed the narrative and major concepts presented in the paper. O.B.D., S.Y., S.P.M., R.N. and Y.P. wrote the manuscript.

## Additional information

**How to cite this article:** Dor, O. B. *et al.* A chiral-based magnetic memory device without a permanent magnet. *Nat. Commun.* 4:2256 doi: 10.1038/ncomms3256 (2013).
